# Depressed-Type Colonic Lesions and “*De Novo*” Cancer in Familial Adenomatous Polyposis: A Colonoscopist's Viewpoint

**DOI:** 10.1155/2013/838134

**Published:** 2013-02-27

**Authors:** Shin-ei Kudo, Yuusaku Sugihara, Hiroyuki Kida, Fumio Ishida, Hideyuki Miyachi, Yuichi Mori, Masashi Misawa, Tomokazu Hisayuki, Kenta Kodama, Kunihiko Wakamura, Takemasa Hayashi, Yoshiki Wada, Shigeharu Hamatani

**Affiliations:** Digestive Disease Center Showa University Northern Yokohama Hospital, Chigasaki Chuo 35-1, Tsuzuki-ku, Yokohama, Kanagawa-ken 224-8503, Japan

## Abstract

Familial adenomatous polyposis (FAP) is the most common inherited polyposis syndrome. Almost all patients with FAP will develop colorectal cancer if their FAP is not identified and treated at an early stage. Although there are many reports about polypoid lesions and colorectal cancers in FAP patients, little information is available concerning depressed lesions in FAP patients. Several reports suggested that depressed-type lesions are characteristic of FAP and important in the light of their rapid growth and high malignancy. Here, we describe the occurrence of depressed-type lesions in FAP patients treated at our institution. Between April 2001 and March 2010, eight of 18 FAP patients had colorectal cancers. Depressed-type colorectal cancer was found in three patients. It should be kept in mind that depressed-type lesions occur even in FAP.

## 1. Introduction

In 1859, Half et al. [1] first described adenomatous polyposis in a 16-year-old female and a 21-year-old male. Later, familial adenomatous polyposis (FAP) was recognized as the most common inherited polyposis syndrome. FAP is an autosomal dominant disease that is classically characterized by the development of hundreds to thousands of adenomas in the rectum and colon during the second decade of life. Almost all FAP patients will develop colorectal cancer if the FAP is not identified and treated at an early stage. Cumulative evidence indicates that, under the umbrella of FAP, classic FAP (cFAP) and attenuated FAP (aFAP) might be very different identities both clinically and molecularly. aFAP is a milder form that is characterized by fewer adenomas and a later age of adenoma development and cancer diagnosis [1].

The genetic basis of FAP is a germline mutation of the adenomatous polyposis coli (APC) gene on chromosome 5 (5q21-q22) [2]. Only about 10%–15% of aFAP cases are secondary to APC. More frequently, in 20%–30% of cases, this condition has been correlated with a biallelic mutation of the MutY human homolog gene (*MUTYH*) located on chromosome 1 (1p34.3-p32.1). Two mutations account for most of the variations of the *MUTYH* gene: Y165C (exon7) and G382D (exon13) [3]. It is difficult to obtain accurate nationwide and global data about the incidence of FAP, although there are local FAP registries in many countries. A 1955 report calculated that the incidence of FAP at birth in the UK was 1 : 8,300 [4].

In classic FAP, the optimal surveillance interval is two years between sigmoidoscopies. If adenomas are detected, colonoscopic investigations should be performed annually until a colectomy is planned. In high-risk individuals (i.e., first-degree relatives of affected patients) from families without an identified APC mutation, surveillance should be continued at two-year intervals before age 40. After this age, the intervals between examinations may be longer (e.g., every 3–5years), and surveillance may be discontinued at age 50.

In aFAP, the optimal surveillance protocol is two years between colonoscopies; since patients with aFAP have been described as having only a few adenomas localized in the right part of the colon, colonoscopy is recommended instead of sigmoid colonoscopy [5].

**Figure 1:**
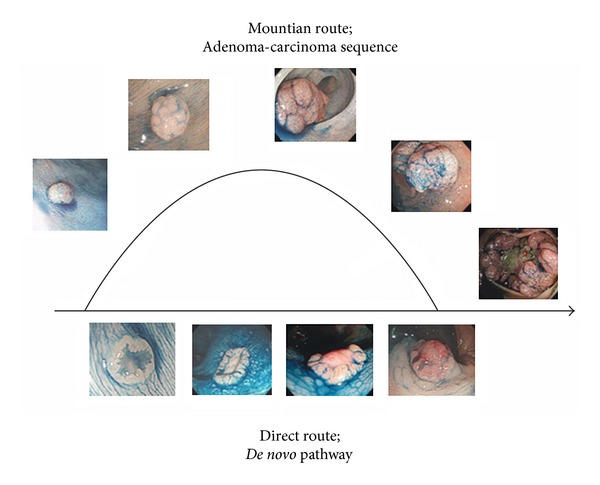
“Adenoma-carcinoma sequence” and “*de novo*” theory regarding the development/progression of colorectal neoplasms. In the adenoma-carcinoma sequence, lesions grow and protrude and finally develop to ulceration; this is called the “mountain route.” Depressed-type lesions develop by the *de novo* pathway. This is called the “direct route.”

**Figure 2:**
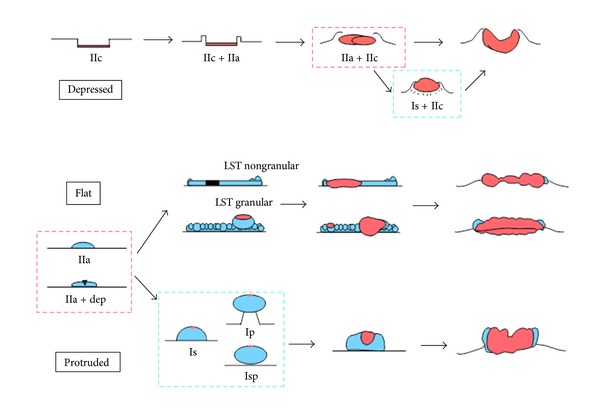
Gross (development/progression) appearance of colorectal neoplasms. This classification is a slight modification of the Paris endoscopic classification and the Japanese rule. The diagnostic characteristics of massive submucosal invasion vary depending on the morphological development of colorectal neoplasms. The red-colored area indicates the cancerous portion.

Concerning the development of colorectal carcinoma, two hypotheses have been advocated; one is the “adenoma-carcinoma sequence” theory [6]. This idea maintains that a cancer develops from normal mucosa through the stage of adenoma. The other hypothesis is the “*de novo*” theory, which claims that a carcinoma emerges directly from normal epithelium without going through a stage of adenoma [7–9] (Figure 1). The adenoma-carcinoma sequence theory had supported that adenomatous polyps have been ulcerated and sloughed away during development. However, most small early cancers without an adenomatous remnant are not ulcerated. Cancers in FAP patients may occur in an adenoma-carcinoma sequence, because many polypoid lesions were observed in the colon. In addition, the diagnostic characteristics of deep submucosal invasion vary depending on the gross appearance (Figure 2). It is not well known that FAP can cause depressed-type lesions as well as polypoid lesions [10].

The depressed-type lesions are sometimes accompanied by massively invasive submucosal cancers. Early detection and timely treatment of the depressed-type lesions are critical for improving the rates of morbidity and mortality due to colorectal cancer. The invasive rate of depressed-type neoplasms is 10.7% in lesions not exceeding 5 mm, 52.6% in those from 6 to 10 mm, and 92.3% in those from 11 to 15 mm. The depressed-type neoplasms are invasive even when they are small (Table 1) [11].

## 2. Aim

We revealed the depressed-type lesions in FAP patients.

## 3. Methods

Between April 2001 and March 2010 at the Showa University Northern Yokohama Hospital, 21 patients with adenomatous polyposis underwent a colonoscopy (FAP, 18 patients; hyperplastic polyposis, 1 patient; Peutz-Jeghers syndrome, 1 patient; Cronkhite Canada syndrome, 1 patient). We analyzed these patients' clinical features by gender, age, and the location, size, and morphology of the colorectal lesions. The gross appearance was evaluated according to the development/progression classification (Figure 2).

## 4. Results

Of the 18 FAP patients, eight were diagnosed when they developed colon cancer at following under progress observation. Seventeen colon cancers were discovered by colonoscopy (Table 2).

Of the eight patients with colon cancer, the gender ratio was one male and seven females; the average age when their colon cancer was detected was 53years (29–78 years). The sites of the colon cancers were cecum, 0; ascending colon, 0; transverse colon, 3; descending colon, 0; sigmoid colon, 7; and rectum, 7. The cancer tended to be on the left side of the colon. The form of the cancer was type0-Isp, five; type0-Isp, two; LST-NG-PD, one. The diagnosis of depth was mucosa, 3; submucosa, 3; MP 3 deeper lesion was 4 lesions.

Notably, three of seventeen lesions (17.6%) were the depressed-lesion type. They were 11-12 mm in diameter. A histopathological examination revealed that these lesions did not have adenoma; their entire contents were adenocarcinoma. The case reports for the patients with these three lesions are as follows.

### 4.1. Case 1

A 59-year-old woman presented with a positive fecal occult blood test. She had no family history of FAP. She had had a colonoscopy at a clinic, and at that time she was diagnosed with type2 colon cancer at the sigmoid colon. She then underwent a laparoscopy-assisted sigmoidectomy. The pathological examination revealed five lesions that were colon cancer.

**Figure 3:**
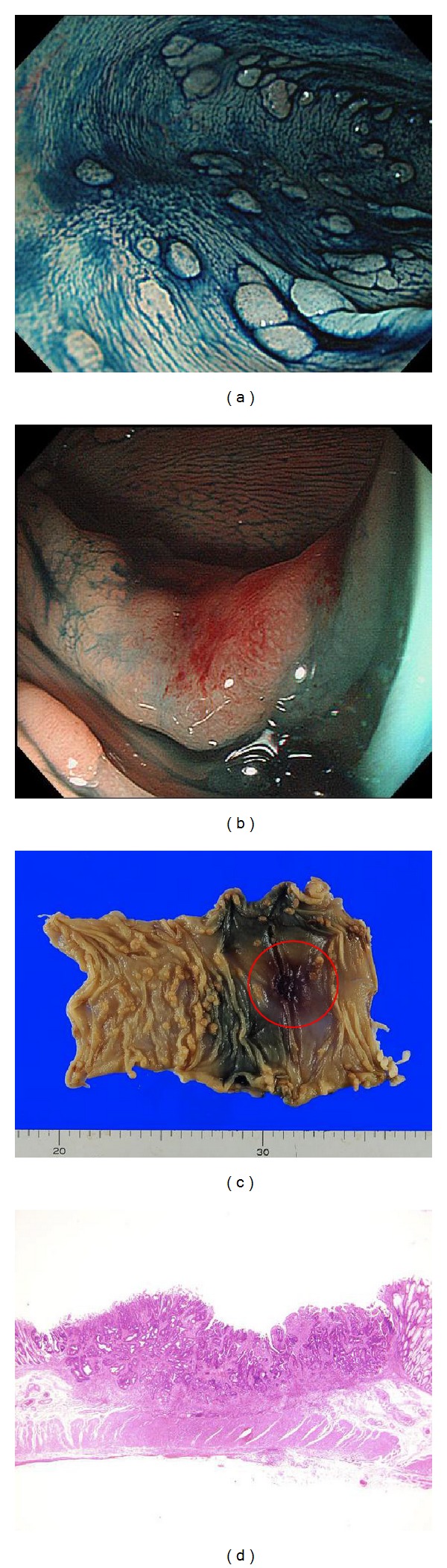
Case 1. (a) Chromoendoscopic image of sigmoid colon after indigo carmine spraying. There were many polyps. (b) The depressed-type lesion at the transverse colon. Magnified view of this lesion. (c) The specimen obtained by laparoscopy-assisted transversectomy. The lesion is within the red circle. (d) The microscopic view of the lesion. This lesion massively invated the submucosal layer. This type0 IIa+IIc cancer had no adenoma.

Two years later, she had a colonoscopy and was diagnosed with aFAP. A depressed lesion was detected at the transverse colon; colorectal cancer was suspected. And then, she received an operation. According to the pathological examination, this lesion was early colon cancer, type0 IIa+IIc, 12 × 9 mm, adenocarcinoma (tub1), SM3 (pSM: 1750 *μ*m), ly1, v1, pN0 (Figure 3).

### 4.2. Case 2

A 65-year-old woman was under observation at her clinic after being diagnosed with FAP. A total colorectomy was recommended, but she refused it and was followedup every six months. A lesion was eventually detected that suggested colorectal cancer at the sigmoid colon. The lesion was IIa+IIc type colon cancer, and the endoscopic diagnosis was submucosal-invasive cancer.

**Figure 4:**
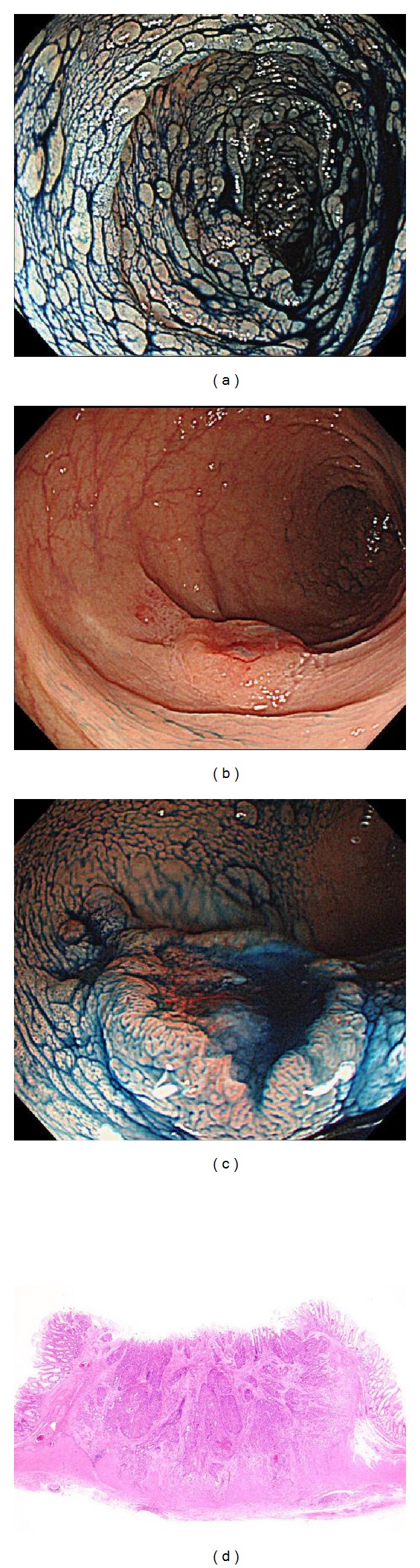
Case 2. (a) Chromoendoscopic image of sigmoid colon after indigo carmine spraying. (b) The depressed-type lesion at the sigmoid colon. (c) Magnified indigo carmine-sprayed image. It was clear that this lesion had a depressed field. (d) A microscopic view of the lesion. This type0 IIa+IIc lesion invaded the muscle layer. The pathology examination revealed no adenoma.

A pathological examination indicated that the lesion was advanced colon cancer, type0 IIa+IIc, 11 × 7 mm, adenocarcinoma (tub2), pMP, ly2, v1, pN1, pPM0 (68 mm), pDM0 (93 mm). No adenoma component was observed in this lesion (Figure 4).

### 4.3. Case 3

**Figure 5:**
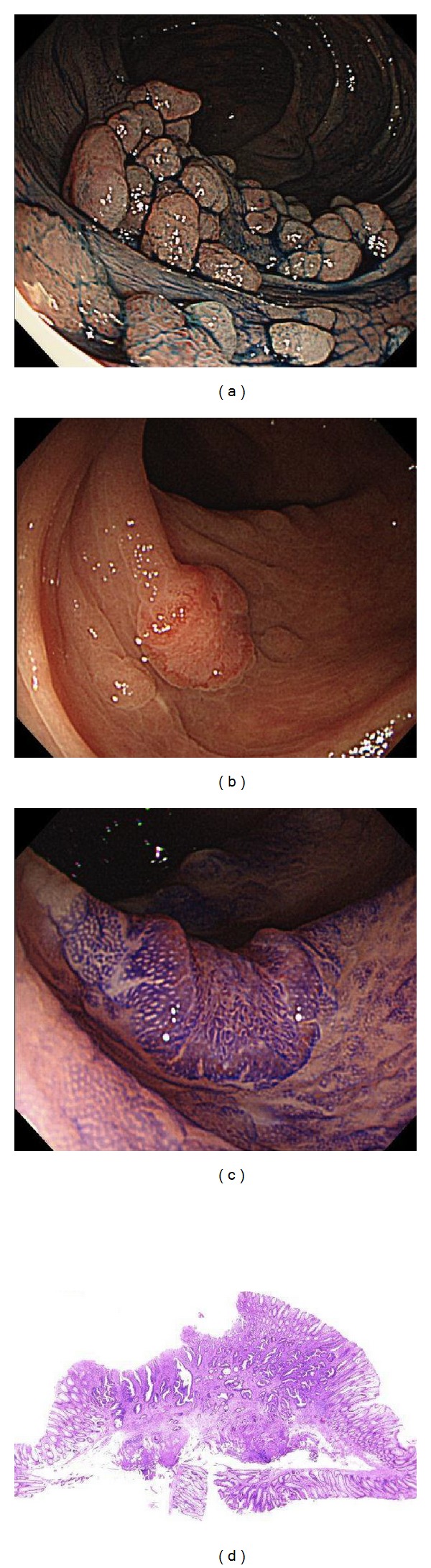
(a) Chromoendoscopic image of transverse colon after indigo carmine dying. (b) The depressed-type lesion at the transverse colon. (c) Magnified image stained by 0.02% crystal violet. The typeV_I_ pit pattern is observed in the depressed surface, and a type I pit is seen at the surrounding margin. (d) The microscopic view of the lesion. This lesions had massively invated the submucosal layer.

A 78-year-old man presented blood feces and was introduced to our institution. A colonoscopy revealed a IIa+IIc lesion at the transverse colon, and the diagnosis of FAP was made. Because the patient declined to undergo surgery, an endoscopic membrane resection was performed. The pathological examination showed that the lesion was well-differentiated adenocarcinoma that had invaded the submucosal layer (Figure 5).

## 5. Discussion

At our institution, we encountered 18 patients with FAP over the course of a decade. Of them, eight patients (17 lesions) were eventually diagnosed with colorectal cancer. Three depressed-type lesions were found among the 17 cancers (17.6%).

During the same period at our institution (from April 2001 to March 2012), a total of 17,291 colorectal cancers—excluding advanced cancers—were resected endoscopically or surgically. Of these, there were 252 depressed lesions (1.4%). 

The adenoma-carcinoma sequence theory was first advocated by Morson in 1968, and it was supported by the genomics study of Vogelstein [11]. The adenoma-carcinoma sequence was considered to be the main route of colorectal cancers. The hypothesized *de novo* pathway is another colorectal cancer pathway. In this theory, *de novo* cancers were described in FAP patients who were thought to have an APC variation. Thus, it might be suggested that *de novo* cancers are also related to a variation of APC genetics.

## 6. Conclusion

We have reported the depressed-type lesions diagnosed in FAP and aFAP patients at our institution. Depressed-type lesions are known to have a high grade of malignancy. Since the lesions were identified in the colons of FAP and aFAP patients, the potential existence of depressed-type lesions should be borne in mind during FAP patients' colonoscopies. More depressed-lesion cases from a variety of institutions should be studied to clarify this phenomenon, and genomics research will also help elucidate the etiology of depressed-type lesions in FAP patients. 

## Figures and Tables

**Table 1:** The invasive rates according to morphology.

Invasive rate according to size distribution						
Size (mm)	–5	6–10	11–15	16–20	21–	Total
Depressed	6/56 (10.7%)	40/76 (52.6%)	60/65 (92.3%)	34/34 (100%)	19/21 (90.4%)	159/252 (63.1%)
Flat	0/3561 (0%)	19/1353 (1.4%)	39/524 (7.4%)	48/364 (13.2%)	144/796 (18.0%)	250/6598 (3.8%)
Protruded	1/4167 (0.02%)	63/4310 (1.4%)	105/1066 (9.8)	91/490 (18.5%)	91/408 (22.3%)	351/10441 (3.3%)

April 2001 to March 2012.

**Table 2:** The characteristics of FAP patients (*n* = 18) treated at our institution between April 2001 and March 2010.

Age	Sex	Location	Type	Size (mm)	Treatment	Depth	Lymph	Vein	Lymph node metastasis
29	F	T	Type 3		Surgery	SI	ly1	v2	N0
44	F	R	Type 2	50	Surgery	MP	ly0	v0	N0
		RS	Type 2	60	Surgery	SE	ly1	v1	N2
59	F	S	Type 2	35	Surgery	SS	ly1	v1	
		S	Type 1	75	Surgery	M			
		R	Type 2	50	Surgery	M			
		S	Is	10	Surgery	SM3	ly1	v1	N0
		R	Is	10	Surgery	MP	ly2	v1	N1
64	F	T	IIa + IIc	12	Surgery	SM2	ly1	v1	
65	F	S	IIa + IIc	11	Surgery	M	ly0	v0	
78	M	T	IIa + IIc	11	EMR	M	ly0	v0	
64	F	R	LST-NG-PD	10	EMR	M	ly0	v0	
29	F	R	Isp	10	Surgery	M	ly0	v0	N0
		R	Is	8	Surgery	M	ly0	v0	
		S	Isp	9	Surgery	M	ly0	v0	N0
		S	Is	10	Surgery	M	ly0	v0	
		S	Is	15	Surgery	SM1	ly0	v0	

A total of 17 cancers were detected in 8 patients during the surveillance period.

Three of these were of the depressed type.

Location abbreviation: T; transeversecolon R; rectum S; sigmoidcolon EMR: endoscopic mucosal resection.
